# Underwater Electromagnetic Sensor Networks, Part II: Localization and Network Simulations

**DOI:** 10.3390/s16122176

**Published:** 2016-12-17

**Authors:** Javier Zazo, Sergio Valcarcel Macua, Santiago Zazo, Marina Pérez, Iván Pérez-Álvarez, Eugenio Jiménez, Laura Cardona, Joaquín Hernández Brito, Eduardo Quevedo

**Affiliations:** 1 Escuela Técnica Superior de Ingenieros de Telecomunicación (ETSIT), Universidad Politécnica de Madrid (UPM), Av. Complutense 30, 28040 Madrid, Spain; javier.zazo.ruiz@upm.es (J.Z.); sergio@gaps.ssr.upm.es (S.V.M.); marina.perez@isom.upm.es (M.P.); 2 Instituto para el Desarrollo Tecnológico y la Innovación en Comunicaciones (IDeTIC), Universidad de Las Palmas de Gran Canaria (ULPGC), 35017 Las Palmas, Spain; eugenio.jimenez@ulpgc.es; 3 Plataforma Oceánica de Canarias (PLOCAN), Telde, 35200 Las Palmas, Spain; laura.cardona@plocan.eu (L.C.); joaquin.brito@plocan.eu (J.H.B.); eduardo.quevedo@plocan.eu (E.Q.)

**Keywords:** underwater communications, ad hoc networks, radio-frequency, localization, Castalia

## Abstract

In the first part of the paper, we modeled and characterized the underwater radio channel in shallow waters. In the second part, we analyze the application requirements for an underwater wireless sensor network (U-WSN) operating in the same environment and perform detailed simulations. We consider two localization applications, namely self-localization and navigation aid, and propose algorithms that work well under the specific constraints associated with U-WSN, namely low connectivity, low data rates and high packet loss probability. We propose an algorithm where the sensor nodes collaboratively estimate their unknown positions in the network using a low number of anchor nodes and distance measurements from the underwater channel. Once the network has been self-located, we consider a node estimating its position for underwater navigation communicating with neighboring nodes. We also propose a communication system and simulate the whole electromagnetic U-WSN in the Castalia simulator to evaluate the network performance, including propagation impairments (e.g., noise, interference), radio parameters (e.g., modulation scheme, bandwidth, transmit power), hardware limitations (e.g., clock drift, transmission buffer) and complete MAC and routing protocols. We also explain the changes that have to be done to Castalia in order to perform the simulations. In addition, we propose a parametric model of the communication channel that matches well with the results from the first part of this paper. Finally, we provide simulation results for some illustrative scenarios.

## 1. Introduction

Underwater wireless sensor networks (U-WSN) are a promising technology to deal with applications that require offshore monitoring, e.g., environmental quality control, seismic analysis, oilfield monitoring and sea robotics [1]. The wireless underwater communication presents, however, a challenging task that needs to be approached depending on the application under consideration. In these two-part papers, we focus on the use of U-WSN in shallow waters.

There are three main technologies that can be considered for underwater wireless communications, namely acoustic, optical and radio. Each of them presents its own benefits and drawbacks and can be used in different scenarios [2,3].

For instance, acoustic communications are valid for medium range distances (several km [4]), but have limited bandwidth and provide poor performance in shallow waters. Acoustic systems near ports or the coastline are affected by multiple sources of noise, such as industries or nearby boats. Furthermore, multi-path associated with different transmission speeds, reverberation and ambient noise also degrade the communication performance.

Optical communications present ultra-high bandwidth over short distances, but are susceptible to turbidity, particles or marine fouling. Ambient light is another adverse effect that can degrade the performance of this technology in shallow waters [5]. Furthermore, this technology currently requires directional communication, as omni-directional wireless transmissions are under research [6].

Electromagnetic (EM) communications in the low frequency range (i.e., kHz) are not affected by ambient noise nor light and can be used in omni-directional or directional deployments. Thus, it becomes a worth-studying option to establish communication between nodes in shallow waters, such as in lakes, bays, harbors and areas close to the sea shores.

In shallow waters, the medium presents two alternative propagation mechanisms for EM waves: the water-to-air and water-to-seafloor interfaces, e.g., [7,8]. Since these interfaces present less conductivity than seawater, depending on the relative position of the transceivers and receivers with regards to the surface and seafloor, the communication range of the EM communication in shallow water is usually larger than over a homogeneous medium.

In the first part of this paper [9], we performed measurements and characterized the underwater channel for shallow waters in a real setup, described the measurement testbed, provided channel measurements at different frequencies and distances and verified the validity of the results using an EM model of the channel and simulations. In this second part, we focus on analyzing the viability of U-WSN using the mentioned EM propagation mechanisms for localization applications. Although the water medium still has a strong attenuation in the low frequency range, we show that reliable communications are possible in the range of tens of meters, making U-WSN viable for deployment. The research described in this paper is part of a national research project named UnderWorld [10], in which all authors are involved.

Our main contribution in this publication is to use the channel characterization from [9,11,12] to show that it is possible to use EM-based U-WSN for localization and monitoring applications. First, we introduce some application requirements for the deployment of a U-WSN, which include low connectivity, low data rate and robustness against packet loss; Then, we consider two localization problems: self-localization for the nodes that have been randomly located during the network deployment and localization of an underwater unmanned vehicle (UUV) for aiding navigation. We propose new and existing localization algorithms that solve these problems and study them under the specific restrictions of underwater radio networks, which include low connectivity, a low data rate and considerable packet loss. Once we understand the requirements of the proposed algorithms, we simulate a complete U-WSN on a prototypical scenario and study the influence of different parameters. In particular, we consider alternative MAC protocols (unicast with ACK and synchronous and asynchronous broadcast) for node-to-node communications (as required for the proposed localization algorithms) and a standard routing protocol for external monitoring. The network simulations are realistic, in the sense that they include multiple effects that affect the overall performance of the network, including propagation impairments (e.g., noise, interference), radio parameters (e.g., modulation scheme, bandwidth, transmit power) and hardware limitations (e.g., clock drift, transmission buffer).

We perform the network simulations in Castalia [13]. Castalia is a simulator for wireless sensor networks based on the OMNeT++ platform [14] that offers realistic wireless channel and radio models and realistic node behavior. The main reasons for choosing Castalia are its level of realism, speed and flexibility. The speed is achieved because all of the modules are written in C++. The flexibility is at the cost of having no graphical user interface. Indeed, Castalia is a command line simulator, where scenarios and settings are defined through external configuration files. The results are also given in text files, but they can be accessed with convenient parser scripts.

The paper is structured as follows. In Section 2, we propose a parametric model of the underwater channel characterized in Part I of this paper [9], so that it can be efficiently simulated. In Section 3, we introduce two algorithms for localization applications and discuss the communication requirements for the network in these scenarios. In particular, in Section 3.1, a self-positioning algorithm is proposed; while Section 3.2 presents a target localization method for navigation aid. In Section 4, we describe the simulations of a complete U-WSN and obtain some guidelines on the communication schemes for the deployment of a network that addresses the proposed applications.

## 2. Parametrization of the Communication Channel

**Figure 1:**
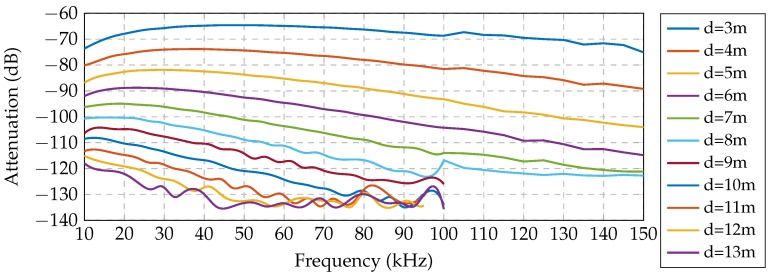
Measurements of the underwater wireless channel: frequency response at multiple distances.

**Figure 2:**
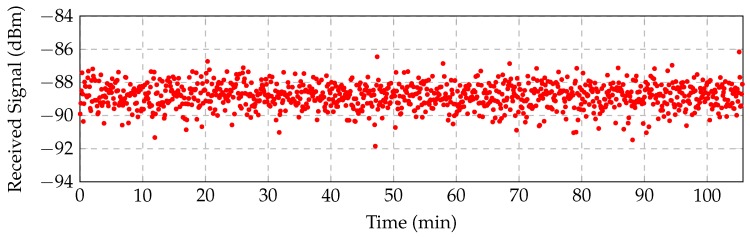
Measurements of the underwater wireless channel: temporal variation at 40 kHz.

We consider a U-WSN consisting of *N* nodes, and we refer to the set of nodes as N={1,…,N}. We performed real measurements in the underwater medium to characterize the channel, where the specific procedure is described in Part I of this paper, presented in [9]. Figure 1 shows the specific signal strengths measured between receiver and transmitter with loop antennas of an 80 cm radius at the seabed. We only show the lowest frequency range (from 10 to 150 kHz), since it offers the lowest attenuation. Figure 2 illustrates the temporal variation and reception noise at 40 kHz along almost two hours. Note also that since the attenuation increases in higher frequencies, the overall bandwidth must be narrow. For the previous reasons, the communication frequencies should be established at a low frequency (∼10 kHz) and present a narrow bandwidth (≤3 kHz).

In Part I of this paper (see [9]), we considered a sophisticated simulator of EM fields to validate the measurement procedure. For the purpose of this paper, we use the EM field simulator to extrapolate the attenuation levels at distances greater than those considered during the measurement campaign. The simulation results appear in Table 1, which require a high computation time. In order to simulate a complete U-WSN with multiple nodes transmitting multiple packets each, we need to express the channel model in a manner that can be simulated with low computational cost. In this section, we propose to approximate the EM model with a parametric path loss model, which basically extends the log-distance path loss model by including a linear term. Let L(d) denote the path loss (dB) at *d* meters from the transmitter. Let $d_{0}$ denote the reference distance (in meters), and let *X* be a random variable that considers random variations in the channel. Then, the proposed model with parameter $\eta_{1}$, $\eta_{2}$ has the following form:

(1) $$L\left(d\right)=L\left(d_{0}\right)+\eta_{1}\frac{d}{d_{0}}+\eta_{2}\;\cdot \;10\;\cdot \;\log\left(\frac{d}{d_{0}}\right)+X$$

We remark that in general, the path loss model should be frequency dependent, but we have assumed the simpler narrow band case. As we will see below, this parametric form provides an efficient characterization of the underwater wireless channel for our network requirements.

In order to find parameters $L(d_{0})$, $\eta_{1}$ and $\eta_{2}$ for the model, we have considered carrier frequency 10 kHz and $d_{0}=3$. Let $x={\left[L\left(d_{0}\right),\eta_{1},\eta_{2}\right]}^{⊤}$ be the vector of unknown parameters. Now, for the parametric model, we introduce the matrix $A=[1,d/d_{0},10\log\left(d/d_{0}\right)]$ of size 14×3, where 1 is a vector of ones. Thus, we have a system of equations, ℓ=Ax, which, using the mean square error (MSE) criterion, can be solved as follows: $x={\left(A^{⊤}A\right)}^{-1}A^{⊤}\ell$, obtaining path loss terms $\eta_{1}=7.2525$, $\eta_{2}=3.6678$ and offset $L(d_{0})=63.0633\;\mathrm{dB}$. The MSE results in 0.3225 dB.

**Figure 3:**
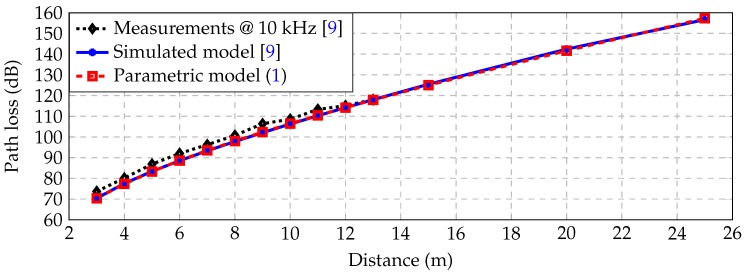
Measurements and path loss models.

Figure 3 shows the real measurements at 10 kHz (black dots) as presented in [9], the simulated EM channel (solid blue) also presented in [9] and the proposed parametric model (dashed red) described by Equation (1). Table 1 presents the measuring distances and the corresponding losses. It is apparent that the parametric model (1) fits the measurements accurately in the given distances.

**Figure 4:**
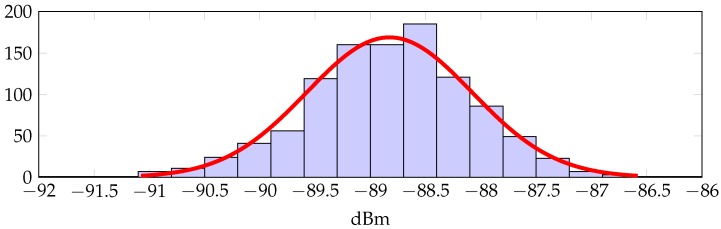
Histogram and normal distribution fit for the trace of measurements.

The additive noise *X* can be also estimated from the trace of measurements displayed in Figure 2. Figure 4 shows the histogram for such a trace together with a normal distribution fit. We conclude that the normal distribution is a reasonable approximation, so that we can assume $X∼N(0,\sigma^{2})$, with $\sigma^{2}=0.56\;\mathrm{dB}$.

A final observation from Figure 2 relevant to the wireless channel simulation is that we can dismiss slow fading.

Once we have fit all of the parameters, we can use the parametric model for estimating the distance between nodes and develop localization algorithms, as explained in the following section.

## 3. Algorithms for Self-Localization and Navigation-Aid Applications

In this section, we develop two localization techniques for implementation in a U-WSN: (i) self-positioning, where nodes cooperate with each other to estimate their position given some reference anchors; and (ii) underwater navigation, where a moving node estimates its position by measuring distances to every node within reach.

We also take into consideration transmission packet losses when solving the localization problems. In Section 4, we will see that the performance obtained when simulating all communication protocols satisfies the requirements derived from the packet-loss analysis of this section.

### 3.1. Self-Localization

We propose a cooperative localization algorithm for nodes that do not know their own position, but can communicate with their neighbors to estimate their own coordinates. We refer to the set of nodes that have knowledge of their position as anchor nodes and denote them with $N_{a}$. Likewise, the set of nodes that do not know their position are denoted as $N_{u}$. We also have $N_{a}\cup N_{u}=N$. We refer to the nodes that are within radio reach of node *i* as neighbors and denote them with $N^{i}$. Note that the set of neighbors may include both nodes from $N_{a}$ and $N_{u}$.

The localization algorithm should be performed only once, when the nodes come online, as an initial calibration step. We analyze its performance in terms of convergence and packet loss. The optimization problem we propose is given by:

(2) $$\begin{array}{c} \min_{x_{i}\in R^{2}}\sum_{i\in N_{u}}\sum_{j\in N^{i}}(d_{ij}^{2}-∥x_{i}-x_{j}{∥}^{2}{)}^{2}=\\ \min_{x_{i}\in R^{2}}\sum_{i\in N_{u}}\sum_{j\in N^{i}}d_{ij}^{4}+∥x_{i}-x_{j}{∥}^{4}-2d_{ij}^{2}{∥x_{i}-x_{j}∥}^{2}. \end{array}$$

Problem (2) is nonconvex and can be solved using algorithms of the family of successive convex approximations, such as the one proposed in [15]. The methodology consists of an iterative procedure where a convex function is used to approximate the non-convex original objective in a local region around a temporary fixed point. The surrogate function retains the convex parts and linearizes the non-convex ones. The convexification is performed around a temporary pivotal point, which is updated in subsequent iteration steps. Specifically, the convex surrogate function for every node $i\in N_{u}$ is given by:

(3) $${\tilde{U}}_{i}\left(x_{i},x^{k}\right)=\sum_{j\in N^{i}}d_{ij}^{4}+∥x_{i}-x_{j}^{k}{∥}^{4}-4d_{ij}^{2}\left(x_{i}^{k}-x_{j}^{k}\right)x_{i}+\frac{\tau }{2}{∥x_{i}-x_{i}^{k}∥}^{2},$$

where $x^{k}={\left(x_{i}^{k}\right)}_{i\in N_{u}}$ is the pivotal point, which is updated at different iteration steps, and τ>0 enforces strong duality. The complete set of instructions to solve Problem (2) is described in Algorithm 1. Note that every node needs to solve the following problem:

(4) $$\begin{array}{cc} {\hat{x}}_{i}\left(x^{k}\right)=\min_{x_{i}} & {\tilde{U}}_{i}\left(x_{i},x^{k}\right) \end{array}$$

where we made it explicit that (4) is minimized only in $x_{i}$ because the algorithm is solved in a distributed manner. Note also that the nodes only need to exchange their own estimates with their neighbors.
**Algorithm 1** Network nodes’ self-localization algorithm1:Initialize
$x^{0}={\left(x_{i}\right)}_{i\in N_{u}}\in R^{2N_{u}}$. Set k←0.2:**repeat**3: For all $i\in N_{u}$, solve $x_{i}^{k+1}\leftarrow {\hat{x}}_{i}\left(x^{k}\right)$.4: Exchange $x_{i}^{k+1}$ with neighbors $j\in N^{i},\;\forall i\in N_{u}$.5: Set k←k+1.6:**until** stopping criteria is satisfied.

Problem (4) is strongly convex and can be solved using any convex optimization solver or, more efficiently, directly finding the solution to the Karush–Kuhn–Tucker (KKT) system of equations [16]. The complexity of this approach is that of finding the root of a strongly monotone function. Efficient methods such as the Newton–Raphson method can be employed to solve it. Although the computation may be more demanding than other approaches in the literature [17], it has the benefit of speeding-up the convergence rate and reducing the required number of messages between nodes. Finally, the algorithm is robust in the sense of converging to a stationary point of the non-convex Problem (2), even when some nodes may have an out of date pivotal point, as analyzed in [18].

### 3.2. Aid to Underwater Navigation

In this section, we consider a different localization algorithm for underwater navigation aid. Underwater navigation is useful in applications where robotic devices or UUVs require orientation to perform some task, such as collecting data from a sink node or exploring the seabed. There are many general localization algorithms in the literature, both distributed and centralized, such as [17,19,20,21]. For the specific application of underwater navigation, the UUV needs to estimate its own position by measuring distances to every node within reach. Since we assume the medium is isotropic, triangulation methods for outdoor localization are sufficient.

We assume that the nodes know their position from a previous calibration, such as the one described in Section 3.1. Then, these nodes transmit a signal to the UUV at a fixed power level together with their local position (if unknown by the UUV). We assume the UUV knows the prefixed transmit power level. Then, the UUV can estimate the distance $\hat{d}$ to each node using the channel form that was described in Section 2. In particular, it needs to solve the following implicit equation:

(5) $$P_{tx}-P_{rx}-L\left(d_{0}\right)=\eta_{1}\frac{\hat{d}}{d_{0}}+10\eta_{2}\log(\frac{\hat{d}}{d_{0}}),$$

where $P_{tx}$ corresponds to the prefixed transmit power level (in dBm); $P_{rx}$ the received power at the UUV (in dBm); and $\eta_{1}$, $\eta_{2}$, $d_{0}$ and $L(d_{0})$ are the channel parameters derived in Section 2. Since the UUV measures all distance estimates directly, it is preferable that it performs all of the computations (i.e., in a centralized manner), so that we avoid the nodes transmitting other data to each other.

Regarding the localization problem, the following optimization problem is frequently proposed:

(6) $$\min_{u}\sum_{i=1}^{n}({\hat{d}}_{i}^{2}-∥u-x_{i}{{∥}^{2})}^{2}$$

where $x_{i}$ represent the network node’s coordinates; *u* the unknown UUV target coordinates; ${\hat{d}}_{i}$ the estimated distances from the sensors to the UUV; and *n* represents the number of measurements available (nodes that can reach the UUV). The localization problem proposed in (6) is static, i.e., it does not consider the speed of a UUV navigating in the network. We remark that in order to consider a dynamic UUV, the model should also take into account the time delay inherent to the underwater communication network.

Problem (6) is non-convex and is also suboptimal in the maximum-likelihood (ML) sense. Nonetheless, since the noise variance is relatively small compared to the channel attenuation (as discussed in Section 2), the solution to the problem yields a good estimate that is close to the theoretical bound of the ML estimator (so that it can be considered accurate enough for many applications). A common formulation that is an alternative to Problem (6) is to obtain the exact ML estimator (see, e.g., [22] Section II-A). Nevertheless, this is a more difficult problem and requires the use of suboptimal methods. It turns out that the algorithms that aim to suboptimally solve the exact ML may yield poorer estimates than the solution of (6), so we do not consider these methods in our simulations. A more thorough discussion can be found in [22].

There are several algorithms in the literature that try to solve (6). For instance, the best linear unbiased estimator proposed in [19] yields a suboptimal result since it solves a relaxed approximation of (6). Other linear estimators, such as the one proposed in [20], minimize an error function derived from (6) rather than the problem itself. Additionally, majorization methods, such as the one proposed in [17], solve the problem by successive convex approximations that converge to local minimization points.

An optimal solution of (6) is however available as presented in [22]. Its solution can be found by reformulating the objective to a non-convex quadratic problem with a single quadratic constraint, which presents strong duality and can be solved optimally [23]. The derivation can be followed in ([22] Section II-B); we summarize it here for completeness. First, we reformulate (6) as an equivalent problem:

(7) $$\begin{array}{cc} \min_{u\in R^{2}} & \sum_{i=1}^{n}\alpha -2x_{i}^{⊤}u+∥x_{i}{∥}^{2}-{\hat{d}}_{i}^{2}\\ s.t. & {∥u∥}^{2}=\alpha . \end{array}$$

By introducing y=(u,α) in Problem (7), we get:

(8) $$\begin{array}{cc} \min_{y\in R^{3}} & {∥By-c∥}^{2}\\ s.t. & y^{⊤}Dy+2f^{⊤}y=0 \end{array}$$

where *B*, *c*, *D* and *f* are defined as follows:

(9) $$B=\left(\begin{array}{cc} -2x_{1}^{T} & 1\\ \vdots & \vdots \\ -2x_{n}^{T} & 1 \end{array}\right),\;c=\left(\begin{array}{c} {\hat{d}}_{1}^{2}-{∥x_{1}∥}^{2}\\ \vdots \\ {\hat{d}}_{n}^{2}-{∥x_{n}∥}^{2} \end{array}\right),\;D=\left(\begin{array}{cc} I_{n} & 0_{n\times 1}\\ 0_{1\times n} & 0 \end{array}\right),\;f=\left(\begin{array}{c} 0_{n\times 1}\\ -0.5 \end{array}\right).$$

The optimization Problem (8) is an instance of a quadratic problem with a single quadratic equality constraint. These kinds of problems have strong duality between the primal and dual formulations provided certain constraint qualifications are satisfied ([23] Th.3.2), which Problem (8) fulfills. Problem (8) can then be solved with the dual formulation:

(10) $$\begin{array}{cc} \underset{\lambda \in R}{\mathrm{solve}} & \hat{y}{\left(\lambda \right)}^{T}Dy\left(\lambda \right)+2f^{T}\hat{y}\left(\lambda \right)=0\\ s.t. & \hat{y}\left(\lambda \right)={\left(B^{T}B+\lambda D\right)}^{-1}\left(B^{T}c-\lambda f\right)\\ & B^{T}B+\lambda D⪰0. \end{array}$$

The objective function of (10) is monotone in *λ*, so a bisection algorithm can be applied and converges very quickly to the unique root of the system [22,23]. The specific steps are described in Algorithm 2.
**Algorithm 2** Bisection algorithm for UUV localization1:Initialize $\underline{\lambda }=-1/\max\mathrm{eig}(D,B^{T}B)$, $\bar{\lambda }=100$.2:**while**
$\hat{y}{\left(\lambda \right)}^{T}Dy\left(\lambda \right)+2f^{T}\hat{y}\left(\lambda \right)>=0$
**do**
$\bar{\lambda }\leftarrow 2\bar{\lambda }$3:**end**
**while**4:Set $\lambda \leftarrow \frac{1}{2}\left(\bar{\lambda }+\underline{\lambda }\right)$.5:**while**
$\bar{\lambda }-\underline{\lambda }\geq \epsilon$
**do**6: **if**
$\hat{y}{\left(\lambda \right)}^{T}Dy\left(\lambda \right)+2f^{T}\hat{y}\left(\lambda \right)>=0$
**then**
$\underline{\lambda }\leftarrow \lambda$7: **else**
$\bar{\lambda }\leftarrow \lambda$.8: **end**
**if**9:**end**
**while**10:Set $\left[u^{T},\alpha \right]\leftarrow {\hat{y}}^{T}\left(\lambda \right)$. **Return**
*u*.

### 3.3. Numerical Experiments

**Figure 5:**
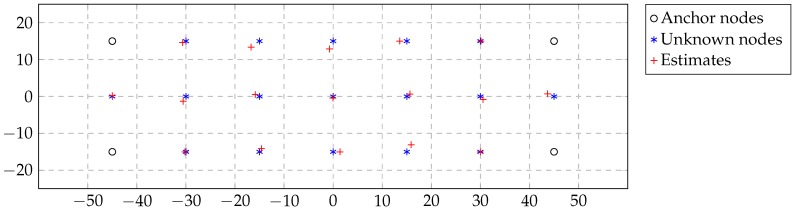
Network self-localization qualitative result of Algorithm 1.

We consider a network of 21 nodes forming a grid, where $|N_{a}|=4$ and $|N_{u}|=17$. The nodes have a separation of 15 m in the horizontal and vertical directions as depicted in Figure 5. We used the channel coefficients derived in Section 2, where the channel model is given by (1). The nodes use 40 dBm of transmit power, as the commercial product [24], and they have an attenuation of −125 dB between two adjacent nodes. We set the reach of two distant nodes at 22 m, which is a reachable distance as explained in Section 4. If the receivers have high sensitivity, about −120 dBm, the signal at such a distance has a strength of −112 dBm and can be detected. This distance allows communication between three neighbors for every node, which makes Problem (2) have a unique solution. Finally, we have considered noise with a standard deviation equal to 2 m for the distance estimation procedure.

**Figure 6:**
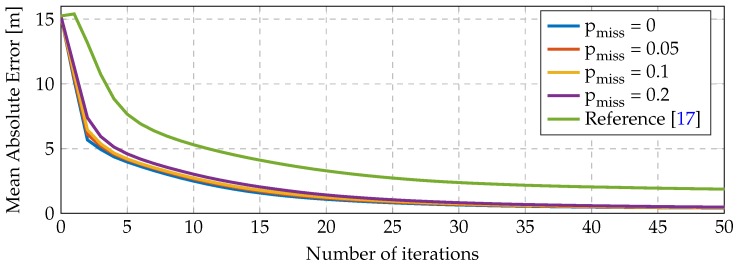
Network self-localization mean absolute error results versus the number of iterations of Algorithm 1. The curves indicate the probability of packet loss of some node to every neighbor.

In Figure 5, we represent the qualitative result of Algorithm 1 in the case where the network does not miss any packet. In Figure 6, we represent the absolute mean error of all nodes with unknown position versus the number of iterations (message exchanges) averaged over 100 simulations. We plot multiple curves corresponding with a 0%, 5%, 10% and 20% probability of packet loss and compare our method with the one proposed in [17]. The algorithm presented in [17] is a deterministic state-of-the-art localization algorithm with distributed computation and low communication requirements, such as the one we propose. Probabilistic methods, such as [25], model the nodes’ position using particle filters and have higher communication requirements, which make them not appropriate for the application at hand.

The packet loss levels are typically caused by different communication impairments, as we show in Section 4. When a packet from some node is lost, Algorithm 1 performs the computation step 4 using the last known estimate of such a node. Figure 6 illustrates that the algorithm is robust against packet losses; in particular, the only effect caused by the packet-loss that can be observed is a slower convergence rate. It also shows a consistent convergence to good estimates of the nodes, whereas the algorithm from [17] converges frequently to local suboptimal solutions, degrading its average performance. For a fair comparison, the same random initialization point was used for the two algorithms.

**Figure 7:**
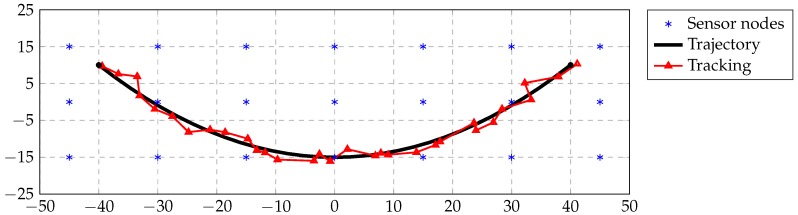
Tracking performance taking into account 22 m of radio sensitivity.

Next, we consider the tracking method proposed in Section 3.2. We plot a qualitative localization result of a target moving in the previous network, which is depicted in Figure 7. The network is the same as in the previous case. We considered that the target node can measure distances from nodes within 22 m. We assumed a random distance deviation of 2 m. The mean absolute error of all points is about 0.96 m. We remark that, although Problem (6) is nonconvex, Algorithm 2 solves Problem (6) in an optimal manner.

The statistical significance of these simulations relies mainly in how well the distances are estimated. The algorithms work very well and are robust to missing packets (mainly due to collisions), but the precision that can be achieved depends mainly on the estimated distances. Channel noise is very low and has little influence on the estimates, but the accuracy in the distance-attenuating curve is critical for proper estimation. From our experiments, we conclude that the parametric model fits the measurements and the EM channel model from [9] very well, but more experiments may be required to confirm this conclusion in a real setting.

## 4. Simulation of a Complete U-WSN

In this section, we simulate a complete U-WSN in the Castalia simulator [13]. The results of this section should be considered realistic, in the sense that our simulations include propagation impairments (e.g., noise, interference between nodes), radio parameters (e.g., modulation scheme, bandwidth, transmit power) and hardware limitations (e.g., clock drift, transmission buffer), and complete MAC and routing protocols have been implemented.

We start discussing the simulation of the parametric channel presented in Section 2. Then, we explain the radio parameters used for the simulations. Finally, we simulate a number of scenarios to obtain relevant figures of merit, like received packet rate or latency. We also explain how to extend Castalia for these simulations.

### 4.1. Simulation of the Parametric Channel

Castalia offers only one parametric channel out of the box, which is the log-distance model. Hence, we had to extend Castalia by including the parametric model (1) into the class WirelessChannel. We have set the parameters found in Section 2: $d_{0}=3\;m$, $L(d_{0})=63.0633\;\mathrm{dB}$, $\eta_{1}=7.2525$, $\eta_{2}=3.6678$ and noise variance $\sigma^{2}=0.56\;\mathrm{dB}$.

Another detail to consider when using Castalia is that it assumes 0 dBm of transmit power by default. Thus, we had to slightly modify the class WirelessChannel to allow for 40 dBm transmit power. In addition, Castalia only sends to each node those radio signals that are above some threshold, so that we had to set the parameter signalDeliveryThreshold = −130 dBm (i.e., smaller than the receiver sensitivity).

### 4.2. Radio Parameters

Modulations are typically classified as bandwidth efficient or power efficient [26]. Bandwidth-efficient modulations, such as high density QAM schemes, are able to accommodate data within a limited bandwidth. Power-efficient schemes, such as BPSK and FSK, are able to reliably send information at the lowest practical power level. In Section 2, we have seen that the underwater medium exhibits high attenuation. Thus, in order to increase the communication range, we require transmitting at high power levels and establishing reliable communication at a low signal to noise ratio (SNR). We chose 2-FSK, since it is power efficient and allows us to use efficient power amplifiers (i.e., FSK provides a constant envelope signal, so that we can use amplifiers that work in saturation mode).

Castalia only offered FSK with a noncoherent receiver and no channel coding. Therefore, we have added the coherent receiver and channel coding to the class Radio (in particular, to the method Radio::SNR2BER). We have chosen a convolutional code (3,1,2), whose non-systematic feedforward encoder is represented by $G\left(D\right)=\left[1+D,\;1+D^{2},\;1+D+D^{2}\right]$, with free distance $d_{\mathrm{free}}=7$ and $B_{d_{\mathrm{free}}}=1$ (this is the total number of nonzero information bits on all weight-$d_{\mathrm{free}}$ paths, divided by the number of information bits per unit time). Let *p* be the bit error rate (BER) obtained from the SNR curve for our FSK modulation. We have characterized the system by including the coding gain as the BER upper bound given by ([27] Equation 12.1):

(11) $$\begin{array}{c} \mathrm{BER}\left(p,B_{d_{\mathrm{free}}},d_{\mathrm{free}}\right)\approx B_{d_{\mathrm{free}}}{\left[2\sqrt{p(1-p)}\right]}^{d_{\mathrm{free}}} \end{array}$$

The radio parameters common to all simulations are: data rate 1 kbps, 1 bit per symbol, signal bandwidth 3 kHz, noise bandwidth 3 kHz, receiver sensitivity −115 dBm, clear channel assessment −112 dBm and noise floor −127 dBm. We assumed carrier frequency separation of 1 kHz.

The required transmit power depends on the scenario under study and influences critically the communication range. Section 4.3.1 shows simulation results for 10 dBm, 25 dBm and 40 dBm, while Section 4.3.2, Section 4.3.3 and Section 4.3.4 only consider 40 dBm. We remark that a transmit power level of 40 dBm is used in commercial products [24]. Castalia has a prefixed value of 0 dBm as the maximum power allowed by the simulator, which must be updated for our setting. In particular, it is needed to increase the value of variable maxTxPower in the WirelessChannel class.

The maximum packet size depends on the application under study. For instance, the localization algorithm considered in Section 3.1 requires small packets of a size of eight bytes (i.e., typically two float numbers per iteration in single precision) before encoding. Recall that the larger the packet, the higher the probability that it suffers some impairment. Section 4.3.1 shows simulation results for random packets of 30 bytes, while the rest of the simulations transmit their neighbor list, which consists of around 15 bytes.

### 4.3. Scenarios under Test

We consider four scenarios: (i) a two-node point-to-point transmission scenario for studying the packet reception rate as a function of the distance; (ii) a network of 21 nodes using diffusion unicast transmissions with acknowledgment (ACK) for studying the ability of an MAC protocol to surmount collisions for different packet periods; since unicast transmissions are costly, in the sense that they require transmitting the same information to every neighbor, we also simulate (iii) diffusion broadcast transmissions (no ACK) in the same network and study the packet rate as a function of a synchronization parameter that ranges from completely asynchronous to a time division multiple access (TDMA) scheme; finally, we consider a (iv) routing in the same 21-node network and study packet rate and latency. For Scenarios (ii)–(iv), all simulations have been performed with a 40 dBm transmit power level and with a coherent receiver with channel coding. All simulation results have been averaged over 50 independent trials.

#### 4.3.1. Point to Point

**Figure 8:**
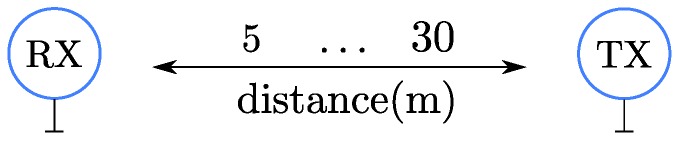
Point to point scenario with two nodes.

The first scenario under study is a two-node, point-to-point setting, where one node transmits and the other listens (see Figure 8).

**Figure 9:**
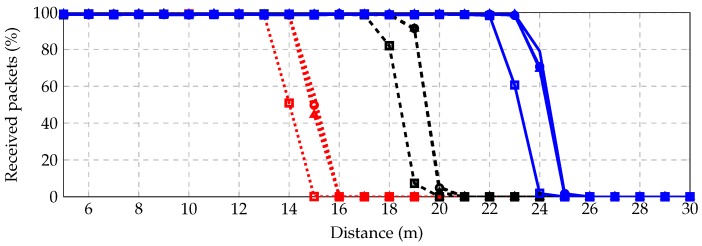
Successfully received packets vs. distance (m).

We study the probability of successfully receiving a packet for different distances between five and 30 m. Figure 9 shows results for three different transmit powers: 10 dBm (red dotted), 25 dBm (black dashed) and 40 dBm (blue solid); and for four different receivers: noncoherent with no channel coding (square), coherent with no channel coding (circle), noncoherent with channel coding (triangle) and coherent with channel coding (no marker). As expected, including a coherent receiver and some channel coding improves the rate. We conclude that with the current radio system, the maximum reliable distance (with 99% success packet reception rate) is around 14 m for 10 dBm, 18 m for 25 dBm and 23 m for 40 dBm.

#### 4.3.2. Diffusion Unicast Transmission with ACK

**Figure 10:**
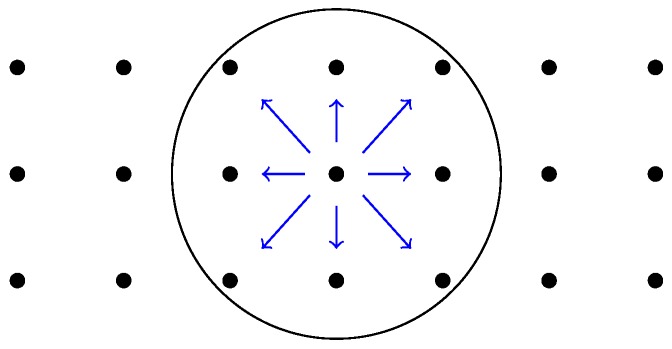
Diffusion scenario.

In this scenario, we simulate a complete network of 21 nodes, which are deployed over a field of $105\;m\times 30\;m=3,150\;m^{2}$ in a three-row by seven-column grid, so that contiguous and diagonal nodes in the same row or column are separated 15 m and 21.2 m, respectively; see Figure 10. The nodes only communicate with their neighbors (see Figure 10), as if they were performing an iterative distributed algorithm (like those presented in Section 3.1).

We use acknowledged unicast transmissions. This means that every node has to discover its neighbor nodes. We use the collection-tree-protocol (CTP) [28,29,30], since it offers an MAC with an ACK layer and an efficient mechanism for neighborhood discovery. CTP has been implemented in several operating systems (e.g., TinyOS, Mantis OS, Contiki OS, Sun SPOTs). It is also offered by Castalia [31] as a set of routing and MAC modules. We remark that for the Castalia implementation of CTP, the neighborhood discovery mechanism is only available for routing tasks. Therefore, we have extended the current CTP implementation so that this neighborhood discovery mechanism can be used for diffusion, as well. We followed the following steps to extend CTP:

1. Create an extra field in the routing packet that accounts for the address of the destination neighbor.
2. Extend CtpForwardingEngine::encapsulatePacket to account for the new field.
3. Include in CtpForwardingEngine::event_SubReceive_receive the case where the node that receives the packet is the destination neighbor, so that it passes the packet to the application layer instead of forwarding it to its parent.

This extension should be also useful for simulating more complex scenarios, where nodes perform a distributed algorithm and also transmit data to the sink, combining autonomous and external monitoring.

When using unicast transmissions, the nodes have to transmit and receive as many packets as neighbors per iteration. In order to minimize congestion, we have modeled an asynchronous algorithm in which, at every iteration, every node transmits its packet to all of its neighbors, waiting some time (named unicast period) between each unicast transmission (i.e., the waiting time before transmitting the same information to the following node in its neighbors’ table). Then, once the current packet has been transmitted to all of its neighbors, each node waits some extra time (named contention period) before starting another iteration. This contention period allows enough retries so that the packets from the latest iteration can be acknowledged. Thus, the time between consecutive packets for node *i* is given by:

(12) $$\begin{array}{c} T_{i}^{\mathrm{uni}}=t_{u}N_{i}+t_{c} \end{array}$$

where $t_{u}$ is the unicast period, $t_{c}$ denotes the contention period and $N_{i}$ is the number of neighbors of node *i*.

**Figure 11:**
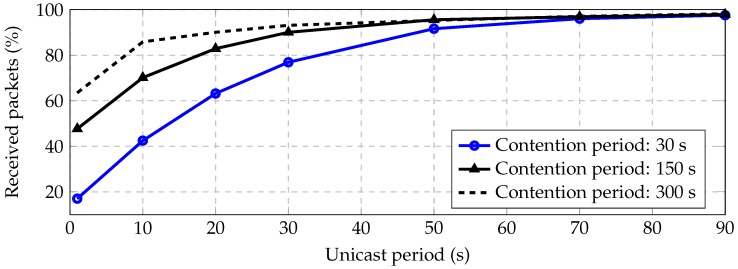
Received packets vs. unicast period for contention periods of 30 , 150 and 300 s.

Figure 11 shows the percentage of successfully received packet vs. unicast period for different contention periods. We have observed that for unicast period 50 s and contention period 30 s, the received packet rate is greater than 91.6%, achieving 98% for 90 s of the unicast period. These long periods are required for low data rates and dense deployments.

We conclude that, although reliable communications are possible using unicast transmissions, it takes a long time to transmit packets safely. The reason is that, since the data rate is low and each node has to transmit the same information to each of its neighbor, the collision probability is high. In addition, multiple collisions imply multiple retransmissions, which also implies high energy consumption, especially when the transmit power is high.

#### 4.3.3. Diffusion Broadcast Transmission (No ACK)

In this section, we consider nodes transmitting to their neighbors over the same setting displayed by Figure 10. Nevertheless, here, we suppose that the nodes broadcast only one packet for all of its neighbors, instead of transmitting one packet per neighbor (this is achieved in Castalia by using the macro BROADCAST_NETWORK_ADDRESS as the destination address).

We study two cases: synchronous and asynchronous transmissions.

**Figure 12:**
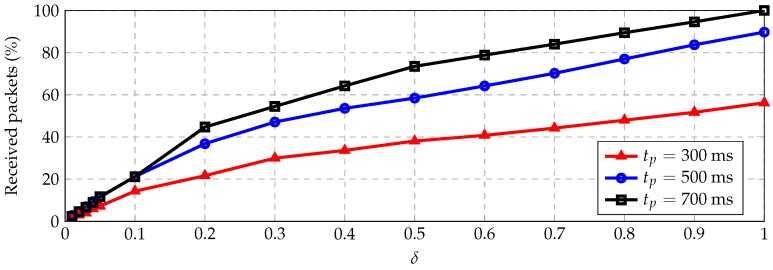
Successfully-received packets vs. asynchronous term *δ* for different waiting times between consecutive packets.

1. Synchronous Transmissions:
  Every node transmits during its own time slot in a time-division-multiple-access (TDMA) manner. Hence, only one node is transmitting at a time, meaning that there is neither interference, nor collisions. The packet loss is only due to channel noise. In order to achieve this TDMA scheme, we have extended the Castalia default application connectivityMap, setting the starting transmission time for node *i* as follows:
  (13) $$\begin{array}{c} T_{i}^{\mathrm{syn}}=Pt_{p}i \end{array}$$
  where $t_{p}$ is the time between consecutive packets from the same node, *P* is the number of packets (in a real algorithm, this should be one, but here, we set P=100 to average the channel noise when estimating the received packet probability) and i∈{1,…,N} is the node identity. Thus, the time that it takes to transmit all packets of all *N* nodes in the network is given by:
  (14) $$\begin{array}{c} T_{\mathrm{net}}^{\mathrm{syn}}=Pt_{p}N \end{array}$$
  Simulations show that we can achieve 99.99% of successfully-received packets by waiting $t_{p}=700\;\mathrm{ms}$ between consecutive packets. This is a relatively high percentage with a minimum number of transmitted packets (neither ACK, nor retries are necessary), but the cost of achieving accurate TDMA synchronization should be taken into account when considering this option.
2. Asynchronous Transmissions:
  By asynchronous transmission, we mean that there is no coordination among nodes. Hence, the packet loss can be due to noise and collisions (i.e., interference). We have parametrized asynchronous transmissions by adding a term δ∈[0,1] to (13), so that the starting time for node *i* becomes:
  (15) $$\begin{array}{c} T_{i}^{\mathrm{as}}=\delta \;\cdot \;T_{i}^{\mathrm{syn}} \end{array}$$
  On the one hand, when δ=0, we have $T_{i}^{\mathrm{as}}=0$ for all i=1,…,N, so that all nodes transmit at the same time (with the exception of very small random clock drifts that can be dismissed for short simulation times). This makes all transmissions collide since no node can receive any packet while it is busy with its own transmission. On the other hand, when δ=1, we have $T_{i}^{\mathrm{as}}=T_{i}^{\mathrm{syn}}$, i.e., we recover the same result as for the perfect synchronization case studied in Section 4.3.3 (1. Synchronous Transmissions). The range 0<δ<1 aims to model the natural level of asynchrony that happens when the nodes transmit at random intervals or when they are initialized at random times. Figure 12 shows the average percentage of received packets vs. the overlapping parameter *δ*.

Since the probability of all nodes choosing the same slot is low, we can expect that the nodes will tend to spread their starting times uniformly (i.e., δ≥0.5); we conclude that asynchronous broadcast transmission is an efficient scheme that allows both a quick packet rate (e.g., $t_{p}=700\;\mathrm{ms}$) and minimum energy consumption (nodes transmit the same packet to all of their neighbors).

#### 4.3.4. Network Routing Using Unicast Transmissions with ACK for Monitoring

**Figure 13:**
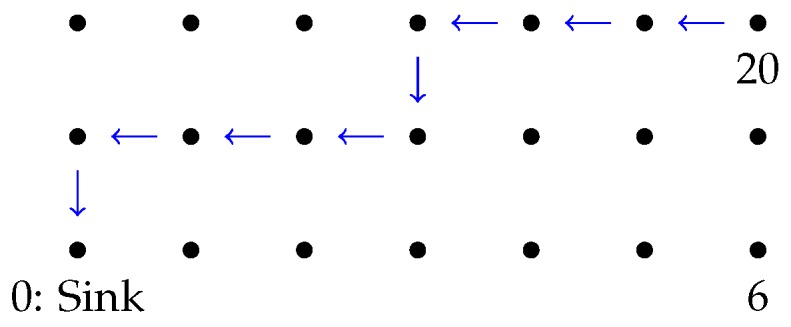
Routing scenario. Example of the path followed by some packet transmitted from the node with identity 20 to the sink. Contiguous nodes are separated 15 m.

In this scenario, we consider the same network topology displayed in Figure 10. Nevertheless, instead of diffusing information within their neighborhoods, the nodes forward packets multiple hops until reaching a sink node; see Figure 13. In this particular experiment, every node sends 50 packets to the sink. Hence, the nodes closer to the sink have to forward multiple packets, apart from transmitting their own packets. The main application for this scenario is to collect data from the network for external monitoring.

We chose the collection tree protocol (CTP) for routing packets to the sink [28,29,30]. CTP uses three techniques to provide efficient and reliable routing: (i) an accurate link estimator that uses feedback from both the data and control planes; (ii) the trickle algorithm [32] to time the control traffic, sending a few beacons in stable topologies, yet quickly adapting to changes; and (iii) actively probing the topology with data traffic, quickly discovering and fixing routing failures. As already mentioned in Section 4.3.2, CTP relies on acknowledged unicast transmissions for estimating the quality of the links.

**Figure 14:**
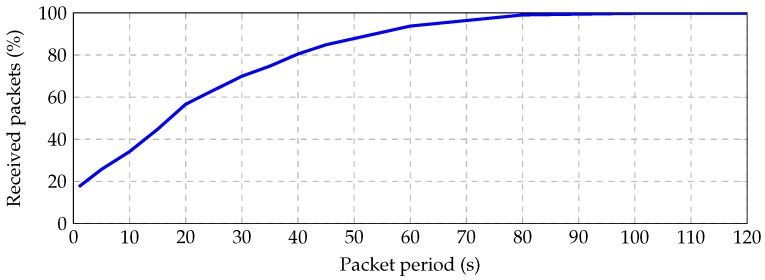
Average successfully routed packets from every node to the sink vs. packet period.

When a node is chosen as the parent from several neighbors or it must perform many retransmissions attempts, its transmission queue can be filled up with unsent packets, and further, incoming packets are dropped. Since the dropped incoming packets are never acknowledged, the link seems down for its neighbors. This kind of congestion becomes worse when nodes operate at low data rates. Although CTP is able to detect and surmount congested nodes, there is some performance limit. We have studied the average number of successfully routed packets from any node to the sink as a function of the packet periodicity. The idea is that the packet period should be long enough for every other node in the network to route its packet to the sink successfully. Figure 14 shows that the application layer should wait 60 s between consecutive transmissions in order to successfully route 93.7% of the packets to the sink, and it must wait 100 s for routing 99.8% of the packets.

**Figure 15:**
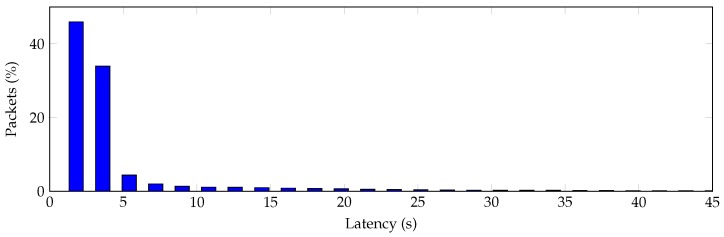
Latency histogram for a packet to be received by the sink.

Another important metric is the time that it takes a packet from any node to be routed to the sink. This latency depends on several issues, like the number of hops in the route, whether there are congested nodes that behave like bottlenecks, packet loss and retries, etc. Figure 15 shows a latency histogram. We have observed that, for this particular scenario and packet period equal to 100 s, 46% of the received packets have latency lower than 1.8 s, 80% lower than 3.6 s, 90.8% lower than 14.4 s and 98.6% lower than 72 s.

In order to perform these simulations, we extended the default CtpTest application such that the sink could store the packet number and source ID for each received packet. This way, we were able to discharge duplicated packets (due to retransmission) and count only the packets that were successfully routed. In addition, we increased the ACK wait delay constant to 0.469 s (by setting the constant CC2420_ACK_WAIT_DELAY = 15360 jiffies) in the CC2420Mac header file to work better with our low bit rate.

Moreover, we set the transmit power to 40 dBm and used coherent receiver and channel coding, so that we achieved good connectivity and every node could find a route to the sink.

## 5. Conclusions

We intend that this publication serves as proof of concept for a prototype U-WSN deployment in shallow waters with complete communication capabilities. In order to accomplish that, we took real measurements of the underwater channel and characterized an EM channel model in the first part of this paper, i.e., [9]. From these measurements and channel model, we fitted a parametric path loss model for narrow-band communications and low noise levels.

We studied and proposed algorithms for self-localization techniques with available neighboring communication. We took into account packet loss considerations due to channel changes and also low connectivity between nodes, as well as low data rate packet limits. We proposed Algorithm 1 for self-localization, which can be solved in a distributed manner, and Algorithm 2 for UUV target localization, which is solved optimally.

We also presented preliminary simulation results of U-WSN in Castalia, a realistic, flexible and fast simulator, the precursor of a real deployment. We considered routing, as well as diffusion scenarios and presented some results that are useful for designing centralized and distributed signal processing algorithms, which are motivated by the previous localization proposals. In order to perform the simulations, we extended Castalia, implementing the proposed path loss model, including coherent receiver and channel coding to the radio and adding diffusion capabilities to CTP.

## Figures and Tables

**Table 1:** Simulated measurements to adjust parametric model (1) as characterized in [9].

Distances (m)	3	4	5	6	7	8	9	10	11	12	13	15	20	25
Path loss (dB)	70	77	83	88	93	97	102	106	110	114	117	125	142	156

## Abbreviations

The following abbreviations are used in this manuscript:

ACK	Acknowledgment
BER	Bit error rate
BPSK	Binary phase-shift keying
CSMA/CA	Carrier sense multiple access, collision avoidance
CTP	Collection tree protocol
EM	Electromagnetic
FSK	Frequency shift keying
KKT	Karush–Kuhn–Tucker
MAC	Multiple access channel
MSE	Mean square error
QAM	Quadrature amplitude modulation
RF	Radio frequency
SNR	Signal to noise ratio
TDMA	Time division multiple access
UUV	Underwater unmanned vehicle
U-WSN	Underwater wireless sensor networks
WSN	Wireless sensor networks
